# Chromosome-scale assembly of the wild wheat relative *Aegilops umbellulata*

**DOI:** 10.1038/s41597-023-02658-2

**Published:** 2023-10-25

**Authors:** Michael Abrouk, Yajun Wang, Emile Cavalet-Giorsa, Maxim Troukhan, Maksym Kravchuk, Simon G. Krattinger

**Affiliations:** 1 Plant Science Program, Biological and Environmental Science and Engineering Division, King Abdullah University of Science and Technology (KAUST), Thuwal, 23955-6900 Saudi Arabia; 2 Center for Desert Agriculture, King Abdullah University of Science and Technology (KAUST), Thuwal, 23955-6900 Saudi Arabia; 3 Persephone Software, LLC, Agoura Hills, CA 91301 USA

**Keywords:** Plant sciences, Genome informatics

## Abstract

Wild wheat relatives have been explored in plant breeding to increase the genetic diversity of bread wheat, one of the most important food crops. *Aegilops umbellulata* is a diploid U genome-containing grass species that serves as a genetic reservoir for wheat improvement. In this study, we report the construction of a chromosome-scale reference assembly of *Ae. umbellulata* accession TA1851 based on corrected PacBio HiFi reads and chromosome conformation capture. The total assembly size was 4.25 Gb with a contig N50 of 17.7 Mb. In total, 36,268 gene models were predicted. We benchmarked the performance of hifiasm and LJA, two of the most widely used assemblers using standard and corrected HiFi reads, revealing a positive effect of corrected input reads. Comparative genome analysis confirmed substantial chromosome rearrangements in *Ae. umbellulata* compared to bread wheat. In summary, the *Ae. umbellulata* assembly provides a resource for comparative genomics in Triticeae and for the discovery of agriculturally important genes.

## Background & Summary

The genus *Aegilops* contains several grass species, commonly referred to as goatgrass. The genus comprises at least 23 diploid and polyploid species and six different genomes (C, D, M, N, S, and U)^1–4^. *Aegilops* species belong to the same tribe as the major cereal crops bread wheat (*Triticum aestivum*, 2n = 6x = 42; AABBDD genome), durum wheat (*Triticum durum*, 2n = 4x = 28; AABB genome) and barley (*Hordeum vulgare*, 2n = 2x = 14). The genus has thus been explored to increase genetic diversity of wheat via wide hybridization and chromosome recombination^5,6^.

**Fig. 1:**
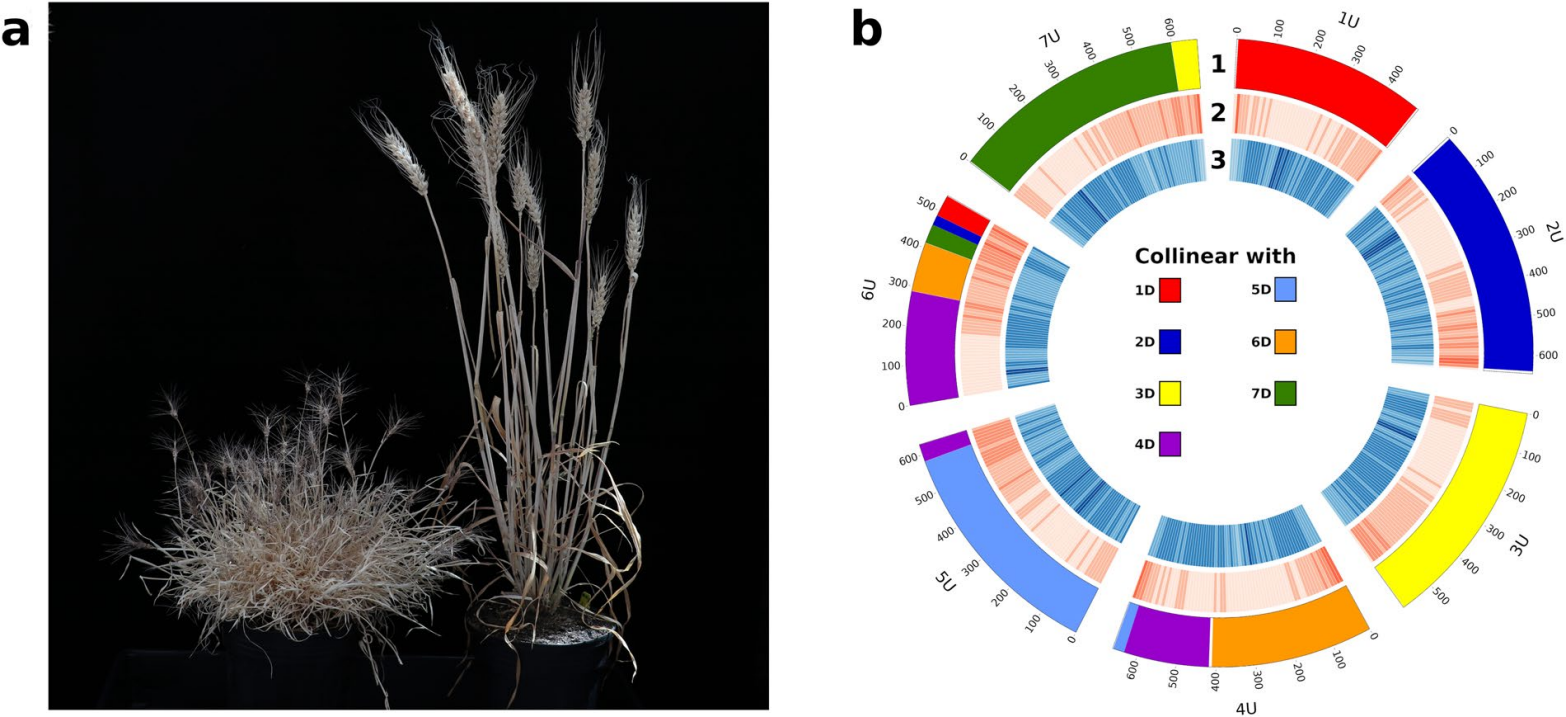
Construction of an *Aegilops umbellulata* chromosome-scale assembly. (**a**) An *Ae. umbellulata* plant (left) is shown next to a bread wheat plant (right) (**b**) Circos plot of *Ae. umbellulata* genome with (1) collinearity blocks against *Ae. tauschii*, (2) gene density and (3) repeat density along each pseudomolecule.

*Aegilops umbellulata* (2n = 2x = 14, UU genome) is the only diploid *Aegilops* species containing the U genome (Fig. 1a). Compared to the bread wheat A, B and D genomes, the U genome contains several large chromosome rearrangements. In particular, chromosomes 4U, 6U,and 7U show multiple reciprocal translocations, inversions and intra-chromosomal translocations^7,8^. The U genome is a source of disease resistance genes that have been transferred into wheat, including *Lr9*, *Lr76*, *Yr70* and *PmY39*^9–11^. Recently, the leaf rust resistance gene *Lr9* has been cloned and found to encode an unusual kinase fusion protein. *Ae. umbellulata* accession TA1851 was identified as the probable donor of *Lr9*^12^. In this previous analysis, a contig-level assembly of TA1851 was generated to evaluate the *Lr9* translocation in bread wheat. The TA1851 contig-level assembly was based on ~157 Gb (~35-fold coverage) of HiFi reads^13^.

In this current study, we first polished the TA1851 HiFi reads using the DeepConsensus^14^ pipeline in order to increase read accuracy and to improve the primary contig-level assembly. We then assembled an *Ae. umbellulata* chromosome-scale reference genome by integrating chromatin conformation capture (Omni-C) data. CpG methylation along the chromosomes was inferred from the PacBio CCS data. The high-quality *Ae. umbellulata* assembly obtained in this study provides a reference for the U genome of the Triticeae tribe. It will serve as the basis to study chromosome rearrangements across different Triticeae species and can be explored to detect U genome introgressions in durum and bread wheat.

## Methods

### Plant material, DNA extraction and sequencing

The DNA extraction and generation of PacBio HiFi reads was described previously^12^. In brief, high molecular weight (HMW) DNA was extracted from young seedlings of *Ae. umbellulata* accession TA1851 using a modified Qiagen Genomic DNA extraction protocol (10.17504/protocols.io.bafmibk6)^15^. DNA was sheared to the appropriate size range (15–20 kb) using Megaruptor 3 (Diagenode) for the construction of PacBio HiFi sequencing libraries. Library preparation was done with the Express Template Prep Kit 2.0 (100-938-900 + Enzyme Clean up 2.0 (101-932-600)), and size was selected with a PippinHT System (Sage Science, HTP0001). Sequencing was performed on PacBio Sequel II systems. The Omni-C library was prepared and sequenced at Cantata Bio using the Dovetail^®^ Omni-C^®^ Kit for plant tissues according to the manufacturer’s protocol. One library was sequenced on an Illumina MiSeq platform to generate ~776 million 2 × 150 bp read pairs for *Ae. umbellulata* accession TA1851.

### Contig-level assembly benchmarking

We first compared contig-level assemblies generated by hifiasm^16^ and the La Jolla Assembler (LJA)^17^ using standard HiFi reads and corrected HiFi reads generated with DeepConsensus^14^. The raw subreads from five SMRT cells were processed using the ccs software (https://github.com/PacificBiosciences/ccs) or DeepConsensus (Table 1). The correction with DeepConsensus produced fewer HiFi data (~157 Gb and ~150 Gb for ccs and DeepConsensus, respectively), but resulted in an increase of the mean read QV (29.9 and 33.1 for ccs and DeepConsensus, respectively) (Table 1).

**Table 1:** Comparison of read quality and yield per SMRT cell between ccs and DeepConsensus pipeline for the generation of HiFi reads.

		Mean read quality	Total bases
SMRT1	ccs	30.2	36,249,624,158
	DeepConsensus	33.2	34,749,641,467
SMRT2	ccs	29	33,632,333,080
	DeepConsensus	32.8	32,111,461,231
SMRT3	ccs	29.8	28,901,671,188
	DeepConsensus	33.2	27,567,325,551
SMRT4	ccs	30.1	29,868,633,741
	DeepConsensus	33.1	28,441,141,874
SMRT5	ccs	30.4	28,845,332,129
	DeepConsensus	33.2	27,540,429,173

Contig-level assemblies generated with the different assemblers and data sets were assessed using the basic summary statistics (Table 2). All four assemblies had similar total assembly sizes. For hifiasm, we observed marked increases of contig N50 (11.1 Mb to 14 Mb; + 26%) and contig N90 (3.2 Mb to 3.8 Mb; + 20%) when using corrected HiFi reads (Table 2). Overall, LJA outperformed hifiasm in terms of contiguity. In comparison to hifiasm, DeepConsensus did not result in a considerable increase of contig N50 with LJA, while the contig N90 increased by 16% (4.5 Mb to 5.2 Mb). The highest contiguity was observed with LJA and DeepConsensus, showing a 59% and 63% increase in contig N50 and contig N90, respectively, compared to the hifiasm assembly with standard HiFi reads (Table 2). In terms of computational resources, all the contig-level assemblies were performed on a single AMD node using 120 cores. We observed that the memory usage was higher with LJA with an increase of 61% and 20% with the standard and corrected HiFi reads, respectively. The computing time was also considerably higher with LJA (Table 2). Based on the overall performance, the LJA-DeepConsensus contig-level assembly was used to construct a chromosome-scale *Ae. umbellulata* assembly.

**Table 2:** Comparison of contig-level assembly metrics between hifiasm and LJA.

	Standard HiFi reads + hifiasm	Corrected HiFi reads + hifiasm	Standard HiFi reads + LJA	Corrected HiFi reads + LJA
Memory used (Gb of RAM)	161.21	149.42	259.57	178.94
Computing time	8 h 27 min	7 h 59 min	45 h 18 min	42 h 38 min
Contig number	1,379	1,521	1,625	1,306
Largest contig (bp)	57,092,498	49,335,673	64,890,551	63,887,064
Total assembly length (bp)	4,254,802,190	4,275,077,199	4,248,511,730	4,246,443,824
N50 (bp)	11,148,243	14,032,818	17,301,094	17,703,042
N90 (bp)	3,182,027	3,817,306	4,472,704	5,187,921
GC (%)	47.1	47.1	47.1	47.1

### Chromosome-scale assembly

Construction of the pseudomolecules was performed by integrating Omni-C read data using Juicer (v2; https://github.com/aidenlab/juicer)^18^ and the 3D-DNA pipeline (https://github.com/aidenlab/3d-dna)^19^. First, to generate the contact maps, Omni-C Illumina short reads were preprocessed with *juicer.sh* (parameters: -s none–assembly). The output file “merged_nodups.txt” and the primary assembly were then used to produce an assembly with 3D-DNA^19^ (using *run-asm-pipeline.sh* with -r 0 parameter). We used Juicebox (v2.14.00)^20^ to visualize the Hi-C contact matrix along the assembly, and to manually curate the assembly. The orientation and the chromosome number of each pseudomolecule were determined based on an existing assembly of *Ae. tauschii*^21^, a close relative of *Ae. umbellulata*, using a dotplot comparison produced with chromeister (https://github.com/estebanpw/chromeister)^22^. There has been some inconsistency in naming the highly rearranged chromosomes 4U and 6U. We decided to follow the most common nomenclature used in the recent publication of Said, *et al*.^8^. Contigs not anchored in the pseudomolecules were concatenated into an “unanchored chromosome”. The final Hi-C contact maps and assemblies were saved using *run-asm-pipeline-post-review.sh* from the 3D-DNA pipeline. The genome assembly resulted in seven pseudomolecules and one unanchored chromosome (Fig. 1b; Table 3).

**Table 3:** Statistics of the *Aegilops umbellulata* pseudomolecule assembly.

Chromosome	Length	Number of contigs	Number of gene models
chr1U_TA1851	494,422,770	44	3,506
chr2U_TA1851	646,201,372	66	5,363
chr3U_TA1851	587,623,253	77	4,444
chr4U_TA1851	663,525,381	83	4,794
chr5U_TA1851	626,841,358	52	5,522
chr6U_TA1851	543,353,244	42	5,075
chr7U_TA1851	664,393,216	66	5,590
chrUn_TA1851	20,213,230	878	1,974

### Repeat annotation and gene model prediction

Transposable element annotation was performed using EDTA^23^ (v2.0.0; parameters: --sensitive 1 --anno 1 --evaluate 1) using the current version of the TREP database (v19)^24^ as a curated input library. Overall, 82.30% of the assembly was classified as repetitive sequences (Table 4).

**Table 4:** Classification of repeat annotation in *Aegilops umbellulata*.

	Class	Count	%masked
LTR	Copia	395,484	17.25%
	Gypsy	1,451,075	34.60%
	unknown	867,939	17.40%
TIR	CACTA	183,488	2.45%
	Mutator	171,834	1.95%
	PIF_Harbinger	90,552	0.95%
	Tc1_Mariner	420,310	3.14%
	hAT	48,882	0.41%
nonTIR	helitron	391,265	4.16%
Total			82.30%

Gene model prediction was performed by combining a lifting approach using liftoff (v1.6.3)^25^ and a genome-guided approach using transcriptomics data with HISAT2 (v2.2.1)^26^, StringTie (2.1.7)^27^ and Transdecoder (v5.7.0)^28^. Post-processing of gff3 files and filtering were performed using AGAT (https://github.com/NBISweden/AGAT)^29^ and gffread (v0.11.7)^30^. For the gene lifting, gene models of hexaploid wheat line Chinese Spring^31^, *Ae tauschii*^21^, and *Triticum monoccocum* accession TA299^32^ were independently transferred using liftoff (parameters: -a 0.9 -s 0.9 -copies -exclude_partial -polish). For the genome-guided approach, we used publicly available RNA-Seq data of 12 representative *Ae. umbellulata* accessions^33^ and the RNA-Seq data of two bulks representing *Ae. umbellulata* leaf tissues^34^. All the RNA-Seq data were mapped individually against the reference sequence using HISAT2 (parameters: --dta --very-sensitive) and the transcripts were assembled using StringTie (parameters: -m 200 -f 0.3) and merged into a single gtf file. The Transdecoder.LongOrfs script was used to identify open reading frames (ORF) of at least 100 amino acids from the merged gtf file. The predicted protein sequences were compared to the UniProt (2021_03) and Pfam^35^ databases using BLASTP^36^ (parameters: -max_target_seqs 1 -outfmt 6 -evalue 1e−5) and hmmer3^37^ (v3.3.2 - parameters: hmmsearch -E 1e-10). The Transdecoder.Predict script was used with the BLASTP and hmmer results to select the best translation per transcript. Finally, the annotation gff3 file was computed using the perl script “cdna_alignment_orf_to_genome_orf.pl” provided in the Transdecoder package.

All the output gff files from the lifting and genome-guided approaches were merged into a single file using the perl script “*agat_sp_merge_annotations.pl*”. The merged file was then post-processed using gffread tools (parameters:–keep-genes -N -J) to retain transcripts with start and stop codons, and to discard transcripts with 1) premature stop codons and/or 2) having introns with non-canonical splice sites. In total, 36,268 gene models were predicted for which the putative functional annotations were assigned using a protein comparison against the UniProt database (2021_03) using DIAMOND^38^ (parameter: -f 6 -k 1 -e 1e-6). PFAM domain signatures and GO were assigned using InterproScan version 5.55–88.0^39^.

The synteny analysis against *Ae. tauschii* was computed using MCScanX^40^ with defaults parameters, which allowed us to identify the main translocation events within the *Ae. umbellulata* genome (Fig. 1b).

### PacBio DNA methylation profile

Methylation in CpG context was inferred with ccsmeth (v0.3.2)^41^, a deep-learning method to detect DNA 5mCpGs by using kinetics features from PacBio CCS reads. The methylation prediction for CCS reads were called using the model “model_ccsmeth_5mCpG_call_mods_attbigru2s_b21.v1.ckpt”. Then, the reads with the MM + ML tags were aligned to the pseudomolecules using BWA (v0.7.17)^42^ and the subsequent BAM file was filtered for hard/soft clips and quality (MAPQ ≥ 60) using SAMtools (v1.8)^43^. The methylation frequency was calculated at genome level with the modbam files and the aggregate mode of ccsmeth with the model “model_ccsmeth_5mCpG_aggregate_attbigru_b11.v2.ckpt”.

### Genome visualization

**Fig. 2:**
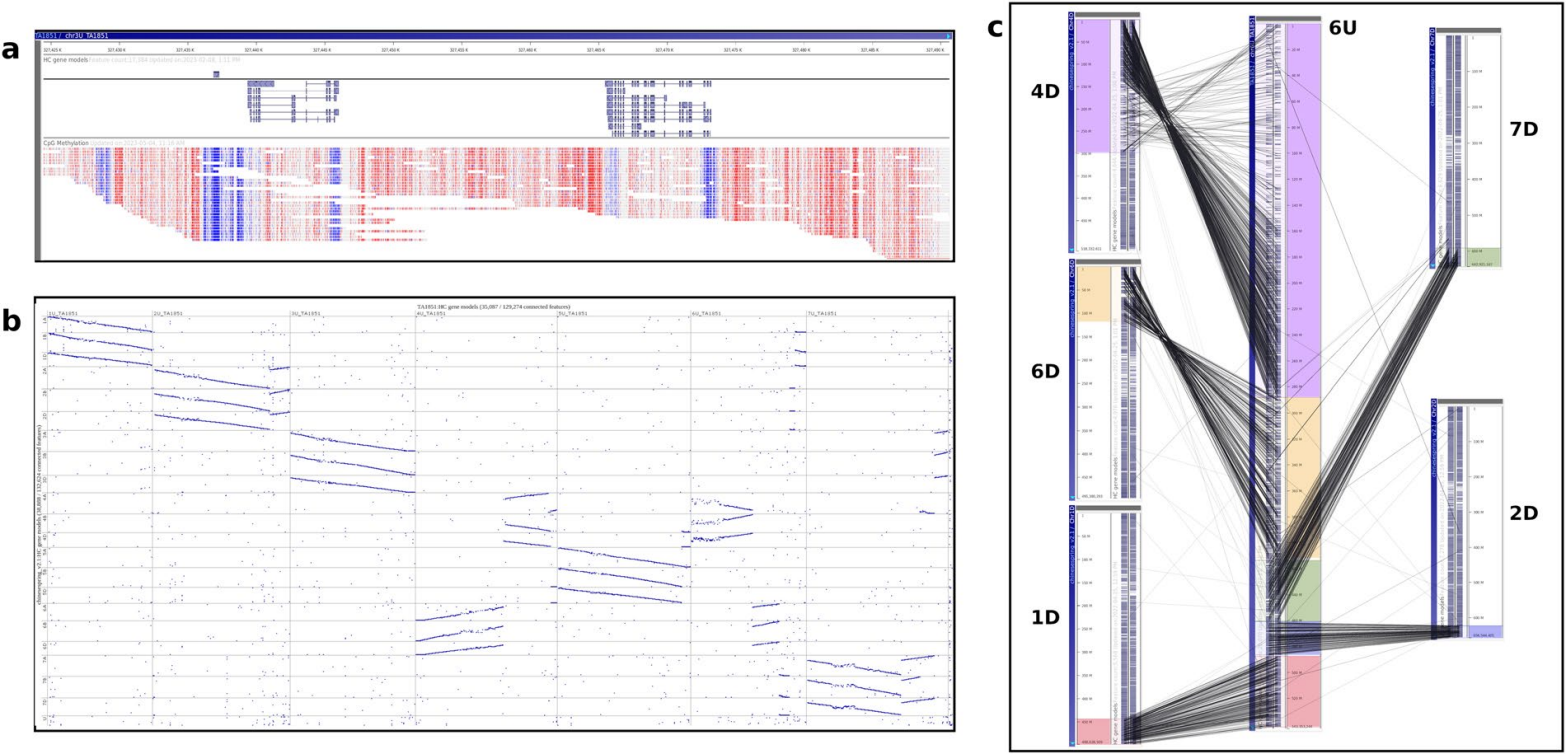
Genome visualization with Persephone. (**a**) Persephone genome browser visualization. The upper panel represents the position along chromosome 3U. The middle panel shows an example of three gene models with their predicted isoforms. In the lower panel, the CpG methylation profile is represented in blue and red for the unmethylated and methylated bases, respectively. (**b**) Synteny matrix between the seven *Ae. umbellulata* chromosomes (x-axis) and the 21 chromosomes of the bread wheat line Chinese spring v2.1 (y-axis) (**c**) Synteny comparison of the highly rearranged *Ae. umbellulata* chromosome 6U (in central position) in comparison to bread wheat chromosomes 1D, 2D, 4D, 6D and 7D. The links between chromosomes represented orthologous gene relationships.

The genome of *Ae. umbellulata* accession TA1851 was uploaded into the Persephone^®^ multi-genome browser (https://web.persephonesoft.com/?data=genomes/TA1851). The data tracks available are the DNA sequence, gene model prediction, and the CpG methylation. A BLAST^36^ search and synteny analysis with the hexaploid wheat line Chinese Spring (v.2.1)^44^ are also available (Fig. 2).

## Data Records

The corrected HiFi reads and the raw Omni-C reads were deposited in the Sequence Read Archive at NCBI under accession number ERP147844^45^. The final chromosome assembly was deposited at NCBI under the accession number GCA_032464435.1^46^.

The *Ae. umbellulata* assembly, gene model prediction, repeat annotations, methylation profile and Hi-C contact map are available on DRYAD Digital Repository^47^ (10.5061/dryad.05qfttf82).

## Technical Validation

### Assessment of genome assembly and annotation

**Fig. 3:**
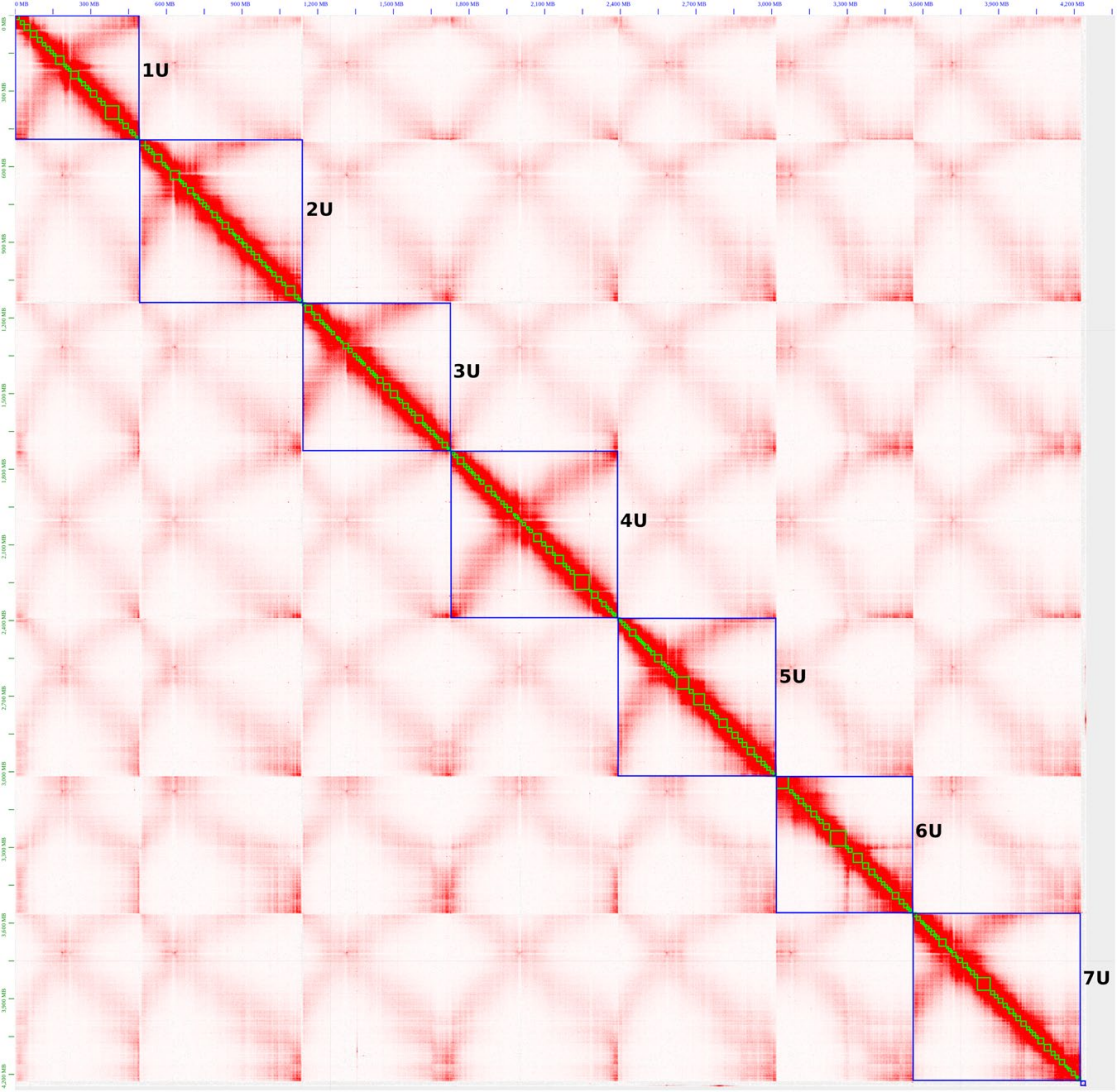
Contact map after the integration of the Omni-C data and manual correction. Green and blue boxes represent contigs and pseudomolecules, respectively.

The Hi-C contact map was manually curated and assessed with Juicebox and showed a dense pattern along the diagonal revealing no potential mis-assemblies (Fig. 3). The anti-diagonals are typical for Triticeae genomes and correspond the Rabl configuration of Triticeae chromosomes^48,49^. Chromosome 6U does not show the anti-diagonal, which is most likely due to the extreme acrocentric nature of this chromosome^50,51^ (Fig. 3).

The BUSCO^52^ (v5.4.5 – poales_odb10) score of 98% (0.4% fragmented and 1.6% missing BUSCOs) at the genome level indicates a high completeness of the TA1851 assembly. The quality of the *Ae. umbellulata* assembly was assessed with Merqury^53^ based on the PacBio HiFi reads using 19-mers. The QV (consensus quality value) and *k*-mer completeness scores were 59.3 and 98.1%, respectively. We further determined the LTR Assembly Index (LAI) and obtained a value of 16.42, which corresponds to a reference quality genome^54^. Telomeric repeats (TTTAGGG)_n_^55,56^ were found at the extremities of all the pseudomolecules, except the short arms of chromosomes 1U and 5U,which corresponds to the location of the rDNA loci in *Ae. umbellulata*^57^.

Completeness of the gene model prediction was evaluated using BUSCO and produced a score of 98.1% (0.3% fragmented and 1.6% missing BUSCOs). The number of predicted gene models (36,268) is in the range of a diploid Triticeae species (34,000–43,000 high-confidence gene models per haploid genome)^58^.

## Data Availability

All software and pipelines were executed according to the manual and protocol of published tools. No custom code was generated for these analyses.
